# Laboratory Investigations in Patients with Community Acquired Sepsis and/or Pneumonia Caused by *Burkholderia pseudomallei*

**DOI:** 10.4269/ajtmh.21-1232

**Published:** 2022-03-28

**Authors:** Bijayini Behera, Anjuna Radhakrishnanan, Baijayantimala Mishra, Prasanta Raghab Mohapatra, Rajesh Kumar

**Affiliations:** AIIMS Bhubaneswar, Microbiology, Bhubaneswar, Odisha, India; E-mail: micro_bijayini@aiimsbhubaneswar.edu.in; AIIMS Jodhpur, Microbiology, Jodhpur, Rajasthan, India; E-mail: anjukrishna008@gmail.com; AIIMS Bhubaneswar, Microbiology, Bhubaneswar, Odisha, India; E-mail: micro_baijayantimala@aiimsbhubaneswar.edu.in; AIIMS Bhubaneswar, Pulmonary Medicine and Critical Care, Bhubaneswar, Odisha, India; E-mail: prmohapatra@hotmail.com; AIIMS Bhubaneswar, General Medicine, Bhubaneswar, Odisha, India; E-mail: drrajeshdr@yahoo.co.in

Dear Sir,

Gassiep and coauthors have made several key observations regarding associations between laboratory investigations and mortality in bacteremic melioidosis cases.^1^ Though there is significant decline in the mortality rates of bacteremic melioidosis cases across various endemic areas attributed to increased awareness, with early diagnosis and optimal management the mortality of bacteremic melioidosis still remains as high as 66%.^2^^,^^3^ As pointed out rightly by the authors, there is limited information available regarding the association of various laboratory parameters at presentation with melioidosis severity and mortality. In recent years, we have witnessed efforts to examine the clinical characteristics of patients with bacteremia caused by specific pathogens.^3^^,^^4^ In this context, we elaborate on the role of laboratory-based investigations in distinguishing septicemic melioidosis cases from other causes of septicemia, in addition to their role as prognostic indicators in melioidosis.

In a prospective study at the All India Institute of Medical Sciences, Bhubaneswar, Odisha State, from July 2018 to March 2020, we identified 33 culture-confirmed cases of melioidosis among a total of 196 patients presenting with community acquired sepsis and/or pneumonia.^5^ We compared laboratory parameters between these 33 culture confirmed melioidosis cases and 163 nonmelioidosis cases presenting with community acquired sepsis and/or pneumonia. Median hemoglobin level (9.5 g/dL [interquartile range (IQR) 4.3–12]) and platelet count (219 × 10^3^/mm^3^, (IQR [19–646]) of melioidosis cases were significantly lower compared with those of the nonmelioidosis cases (10.9 g/dL [IQR 2.4–16.3]), *P* = 0.001 and 309 × 10^3^/mm^3^, ([IQR 50–856], *P* = 0.003). On admission median Sequential Organ Failure Assessment (SOFA) score (4 versus 3, *P* = 0.009) and median quick SOFA (qSOFA) score (2 versus 1, *P* < 0.001) was higher in melioidosis cases compared with nonmelioidosis cases. Out of 33 confirmed melioidosis cases, 7 died (7/33, 21%). Various hematological and biochemical test results and risk factors were compared between the survivors and nonsurvivors; on bivariate analysis, there was no significant association between specific laboratory parameters or risk factors (diabetes mellitus, alcoholism, chronic kidney disease, malignancy, or bacteremia) and mortality.

In our study, higher SOFA or qSOFA and lower median hemoglobin level and platelet count at admission were predictive of melioidosis among patients presenting with community acquired sepsis. In another recent study, median modified SOFA score was higher in patients presenting with *Burkholderia pseudomallei* bacteremia compared with that with *Escherichia coli* and *Staphylococcus aureus* bacteremia.^3^ In our study of a small cohort of 33 culture-confirmed melioidosis cases, we did not find association between specific hematological or biochemical test results or tested risk factors and mortality. Detailed analysis of baseline laboratory parameters stratified by pathogen group should be attempted in large-scale studies to improve the selection of empiric antimicrobial therapy and predict patient outcomes.
